# The Broad Habitat Spectrum of the CL500-11 Lineage (Phylum Chloroflexi), a Dominant Bacterioplankton in Oxygenated Hypolimnia of Deep Freshwater Lakes

**DOI:** 10.3389/fmicb.2018.02891

**Published:** 2018-11-27

**Authors:** Yusuke Okazaki, Michaela M. Salcher, Cristiana Callieri, Shin-ichi Nakano

**Affiliations:** ^1^Center for Ecological Research, Kyoto University, Otsu, Japan; ^2^Bioproduction Research Institute, National Institute of Advanced Industrial Science and Technology, Tsukuba, Japan; ^3^Limnological Station, Institute of Plant and Microbial Biology, University of Zurich, Zurich, Switzerland; ^4^CNR-IRSA Institute of Water Research, Microbial Ecology Group, Verbania, Italy

**Keywords:** bacterioplankton, CL500-11, CARD-FISH, perialpine lakes, oxygenated hypolimnion

## Abstract

CL500-11 (phylum *Chloroflexi*) is one of the most ubiquitous and abundant bacterioplankton lineages in deep freshwater lakes inhabiting the oxygenated hypolimnion. While metagenomics predicted possible eco-physiological characteristics of this uncultured lineage, no consensus on their ecology has so far been reached, partly because their niche is not clearly understood due to a limited number of quantitative field observations. This study investigated the abundance and distribution of CL500-11 in seven deep perialpine lakes using catalyzed reporter deposition-fluorescence *in situ* hybridization (CARD-FISH). Samples were taken vertically (5–12 depths in each lake) and temporally (in two lakes) at the deepest point of the lakes located in Switzerland, Italy, and Austria with varying depth, trophic state, mixing regime, and water retention time. The results showed a dominance of CL500-11 in all the lakes; their proportion to total prokaryotes ranged from 4.3% (Mondsee) to 24.3% (Lake Garda) and their abundance ranged from 0.65 × 10^5^ (Mondsee) to 1.77 × 10^5^ (Lake Garda) cells mL^-1^. By summarizing available information on CL500-11 occurrence to date, we demonstrated their broad habitat spectrum, ranging from ultra-oligotrophic to meso-eutrophic lakes, while low abundances or complete absence was observed in lakes with shallow depth, low pH, and/or short water retention time (<1 year). Together with available metagenomic and geochemical evidences from literatures, here we reviewed potential substrates supporting growth of CL500-11. Overall, the present study further endorsed ubiquity and quantitative significance of CL500-11 in deep freshwater systems and narrowed the focus on their physiological characteristics and ecological importance.

## Introduction

In thermally stratified lakes, the water layer below the thermocline is defined as hypolimnion. In deep lakes this large volume of water is generally oxygenated throughout the year due to (i) winter vertical mixing, (ii) downward intrusion of oxygenated cold water from rivers (Ambrosetti et al., 2010), and (iii) low oxygen consumption due to their oligo- to mesotrophic conditions. In those lakes, the oxygenated hypolimnion accounts for the major parts in volume, and thus, their microbial composition is of significant importance in overall biogeochemical processes.

The CL500-11 lineage (phylum *Chloroflexi*, class *Anaerolineae*) is one of the most abundant microbial groups in oxygenated hypolimnia. After its first discovery by 16S rRNA gene cloning-sequencing as a dominant bacterioplankton lineage in a deep sample (500 m) from Crater Lake (United States) (Urbach et al., 2001), their 16S rRNA gene sequences have been reported from global deep freshwater lakes (Okazaki et al., 2013). Subsequently, catalyzed reporter deposition-fluorescence *in situ* hybridization (CARD-FISH) revealed a dominance of CL500-11 in the hypolimnia of Lake Biwa (Japan, 16.5% of the total bacterioplankton in maximum) (Okazaki et al., 2013), Lake Michigan (United States, 18.1%) (Denef et al., 2016), six deep Japanese lakes (3.9–25.9% in each lake) (Okazaki et al., 2017), and Lake Zurich (Switzerland, 11%) (Mehrshad et al., 2018). Despite their high abundances in deep water layers, CL500-11 were barely detected in the epilimnia during the stratification period, indicating their specificity to the hypolimnion (Okazaki et al., 2013, 2017; Okazaki and Nakano, 2016). Cells of CL500-11 are vibrioid and relatively larger (1–2 μm length) than average prokaryotes in the water column (Okazaki et al., 2013, 2017; Denef et al., 2016; Mehrshad et al., 2018), suggesting their contribution in biomass is even higher than in abundance. Monthly profiles taken for almost 2 years in Lake Biwa revealed a recurrent annual turnover of CL500-11 population, in which their dominance was only observed during stratification and discontinued in the following winter mixing period (Okazaki et al., 2013). This dynamic population succession suggests ecological and biogeochemical significance of CL500-11 in deep lakes’ ecosystems.

The proposed ubiquity and numerical dominance of CL500-11 in the deep oxygenated hypolimnion was recently challenged by CARD-FISH reports of low abundances or even complete absence in several lakes and reservoirs (Okazaki et al., 2017; Mehrshad et al., 2018). However, factors driving their distribution pattern and defining their ecological niche have not yet been clearly understood, although various environmental parameters including the lake trophic state, hypolimnetic temperature, oxygen concentration, and water retention time of the lakes, have been examined (Okazaki et al., 2017). Several metagenome-assembled genomes (MAGs) allowed insights in the eco-physiology of CL500-11, by which they were characterized as aerobic heterotrophs, harboring genes for flagellar motility and xanthorhodopsins, and potentially utilizing nitrogen-rich dissolved organic matter (DOM) and exogenous reduced sulfur compounds (Denef et al., 2016; Mehrshad et al., 2018). However, no consensus on their ecological niche has been reached so far, ultimately owing to a lack of cultivated representatives, but also because of a limited number of quantitative field observations, which is required to understand their lifestyle and interpret metagenomic implications.

The European perialpine lakes are potentially major habitats for CL500-11 since many of them are deep and oligo- to mesotrophic in general with an oxygenated hypolimnion. In fact, 16S rRNA sequences of CL500-11 have been retrieved from Lake Geneva (Humbert et al., 2009) and Lake Zurich (Van den Wyngaert et al., 2011; Mehrshad et al., 2018) which are perialpine lakes. Although CARD-FISH has been commonly employed to quantitatively investigate bacterioplankton communities in perialpine lakes (Salcher, 2013; Salcher et al., 2015; Callieri et al., 2016; Shabarova et al., 2017; Hernández-Avilés et al., 2018; Neuenschwander et al., 2018) quantification of CL500-11 was unprecedented in these lakes, until a recent report from Lake Zurich (Mehrshad et al., 2018). To fill these gaps, the present study aims to measure the abundances of CL500-11 in seven deep perialpine lakes by means of CARD-FISH. The results revealed their broad habitat spectrum and quantitative significance, and together with latest information from literatures, here we review possible eco-physiological characteristics of CL500-11.

## Materials and Methods

### Collection of Water Samples and Environmental Measurements

Water samples were taken at a pelagic station in the deepest area of seven perialpine lakes with a variety of sizes, depths and trophic states (Table 1). Single vertical samples were collected from Lake Como (5 depths), Iseo (5 depths), Garda (5 depths), Thun (9 depths), and Mondsee (12 depths) during summer stratification, while time-series samples were taken from Lake Zurich (9 depths at biweekly) and Maggiore (4 or 5 depths at once or twice a month) to cover the whole stratification period. Samples for cell counting and CARD-FISH were fixed with 2% formaldehyde immediately after the collection and kept at 4°C in the dark until further processing. Total prokaryotic abundance was determined by enumeration of 4′,6-di-amino-2-phenylindole (DAPI) stained cells (Porter and Feig, 1980). The prokaryotic abundances in Lake Maggiore were determined in the integrated 0–20 m (epilimnetic) and 20–350 m (hypolimnetic) as described previously (Bertoni et al., 2010; Hernández-Avilés et al., 2012). Vertical profiles of water temperature, pH, and dissolved oxygen concentration were determined *in situ* with a multiparameter probe at the time of sampling. Chemical parameters of the sampled waters were determined following the standard methods by APHA and AWWA (2005) for Lake Maggiore, Como, Iseo, and Garda, and by the International Organization for Standardization (ISO 13395:1996, 11732:2005, and 15681-2:2003) for the other lakes.

**Table 1:** Limnological characteristics of the study sites.

Lake	Country	Latitude (N)	Longitude (E)	Max. depth (m)	Surface area (km^2^)	Water volume (10^6^m^3^)	Surface elevation (m)	Water retention time (y)	Trophic state	Sampling date
Zurich	Switzerland	47°15′	8°41′	136	88	4300	406	1.4	Mesotrophic	2015.1.7–2015.12.16 (Biweekly)
Maggiore	Italy/ Switzerland	45°48′	8°39′	370	213	37900	193	4.1	Oligo-mesotrophic	2011.3.8–2012.1.12 (Once or twice a month)
Como	Italy	46°00′	9°15′	410	146	22500	198	4.5	Mesotrophic	2011.7.12
Iseo	Italy	45°44′	10°04′	251	62	7600	186	4.1	Meso-eutrophic	2011.7.11
Garda	Italy	45°42′	10°43′	350	368	48900	65	26.6	Oligo-mesotrophic	2011.9.26
Thun	Switzerland	46°41′	7°43′	217	48	6500	558	1.8	Oligotrophic	2010.7.12
Mondsee	Austria	47°50′	13°20′	68	14	520	481	1.8	Mesotrophic	2016.9.14


### CARD-FISH Analysis

CARD-FISH for CL500-11 cells was performed as described by Okazaki et al. (2013) using the same probe CLGNS-584 (5′-GCCGACTTGCCCAACCTC-3′) and helper CLGNS-567h (5′-CTACACGCCCTTTACGCC-3′) set, which have been demonstrated to produce consistent results with the 16S rRNA gene amplicon sequencing analyses (Denef et al., 2016; Okazaki et al., 2017). The specificity of the probe was reconfirmed using the TestProbe 3.0 tool^1^ against the SILVA (Quast et al., 2013) SSU 132 Ref NR99 database (includes only non-redundant, high-quality, nearly full-length 16S rRNA gene sequences). The result indicated that the two CL500-11 16S rRNA gene sequences in the database (accession numbers = AF316759 and HM8566384) were exclusively targeted by the probe. Further, inspection against the SILVA SSU 132 Parc database (includes all available sequences containing relatively shorter and lesser quality ones) demonstrated that all the 60 probe-targeted sequences in the database were closely related to AF316759 or HM8566384 (identity > 97%; Supplementary Table S1), except one sequence (identity = 95.9%) that presumably contains erroneous bases as it is a raw output from the error-prone 454 sequencing platform (KM133681). These results collectively support the specificity of the probe CLGNS-584 in the latest 16S rRNA gene database.

The hybridization was performed at 35°C and the optimum formamide concentration in the hybridization buffer was determined as 40% by testing a series of formamide concentration (5% interval) to obtain the best stringency (that is, highest concentration without signal loss). A previous study that separately investigated the optimum formamide concentration for the same probe also supported 40% to archive the best stringency (Mehrshad et al., 2018). At least 1000 DAPI-positive cells and corresponding FISH-positive cells were counted in each sample using an automated high-throughput microscopy (Zeder and Pernthaler, 2009), which has been widely applied to quantifications of FISH-positive bacterioplankton cells in freshwater systems (Salcher et al., 2011, 2015; Shabarova et al., 2017; Neuenschwander et al., 2018). The abundance of CL500-11 were calculated by multiplying their relative proportions (determined by CARD-FISH) with the total prokaryotic abundances (determined by DAPI-counts).

### Data Analysis

Chemical measurements were collected from literatures for lakes where CARD-FISH on CL500-11 has been carried out previously. However, not every parameter was available in all lakes. To compare values among the lakes, the maximum and minimum of total nitrogen and phosphorus recorded in each lake were used. The relationships between CL500-11 abundance (the maximum value recorded in each lake) and limnological or chemical properties of the lakes were statistically inspected by the Spearman’s rank test using the R 3.4.3 software (R Core Team, 2017). The values used for the analysis and the sources for the data are summarized in Supplementary Table S2.

## Results and Discussion

**FIGURE 1:**
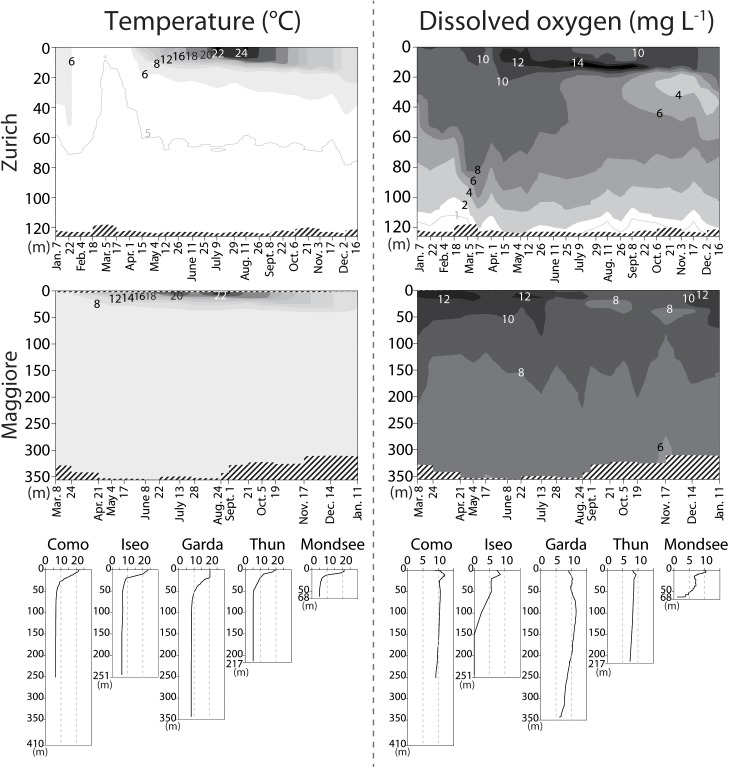
Profiles of the temperature (left panels; °C) and dissolved oxygen (right panels; mg L^-1^) measured *in situ*. The vertical axes indicate water depth. For Lake Zurich and Maggiore, the sampling dates are indicated in the horizontal axes, and spatio-temporal measurements are shown by contours. Shaded areas indicate missing measurements.

**FIGURE 2:**
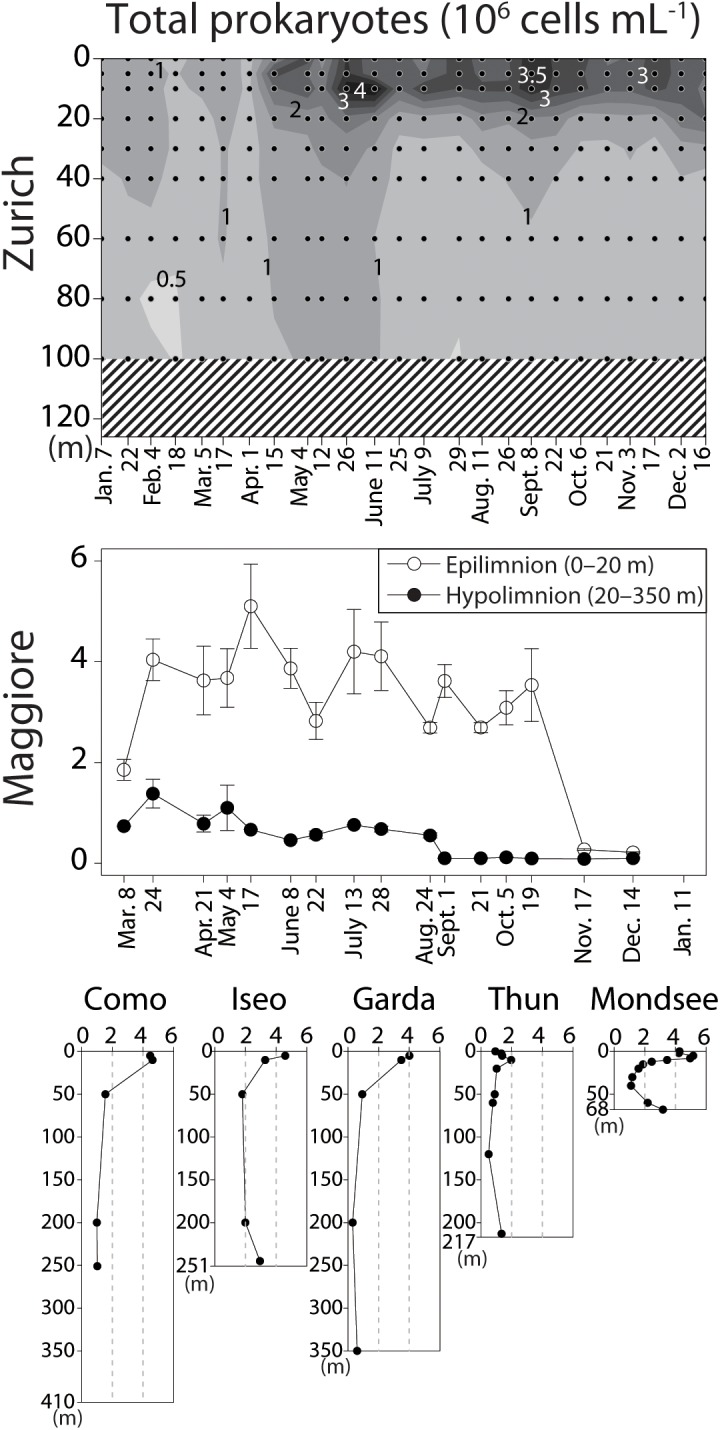
Total prokaryotic abundance determined by enumeration of DAPI-stained cells (10^6^ cells mL^-1^). The vertical axes indicate water depth. For Lake Zurich, spatio-temporal measurement is shown by contours. Dots indicate sampling depths and dates, shaded areas missing measurements. The data for Lake Maggiore were measured in an integrated sample for epilimnetic (0–20 m) and hypolimnetic (20–350 m) water (see main text for detail). Error bars indicate standard error.

**FIGURE 3:**
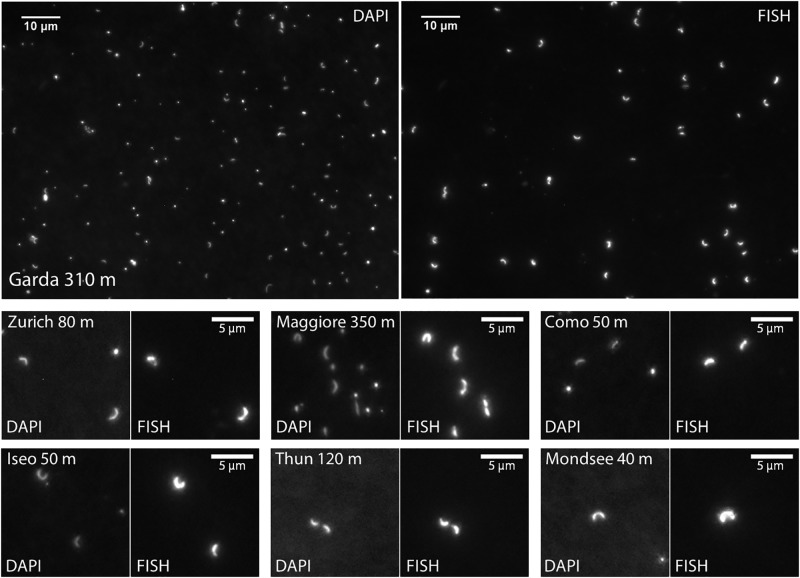
Microscopic images of CL500-11 cells detected by CARD-FISH. In each panel, DAPI-stained cells and the corresponding FISH-positives were shown in the left and right side, respectively. A whole microscopic field was shown for the sample taken from 310 m in Lake Garda, where the highest proportion of CL500-11 cells was recorded. For the other lakes, enlarged images were shown. The samples taken on December were used for the images from Lake Zurich and Maggiore.

**FIGURE 4:**
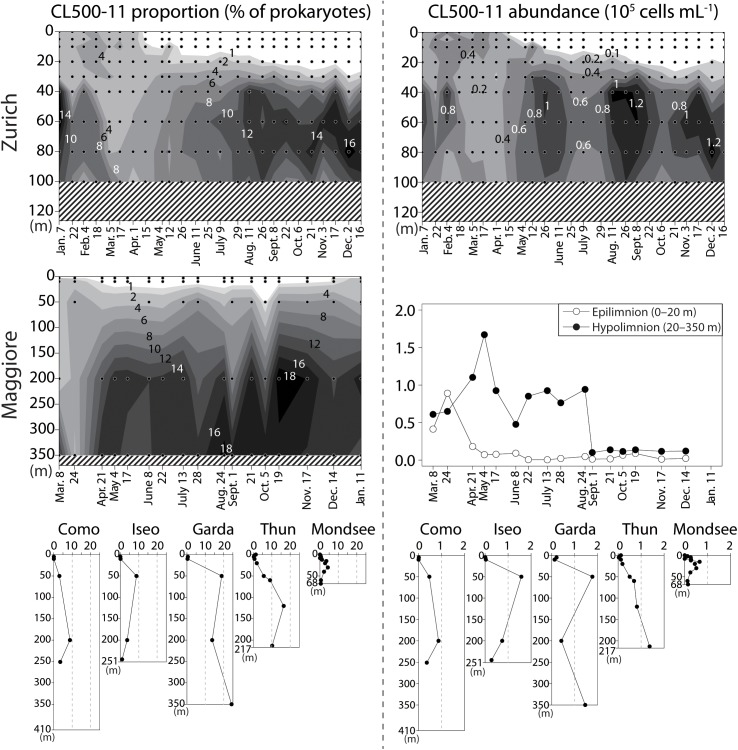
Proportions of CL500-11 in percentage of total prokaryotes (FISH-positive vs. DAPI-stained cells) (**left**; %) and their absolute abundances (**right**; 10^5^ cells mL^-1^) generated by multiplying relative proportions with total prokaryotic abundances (Figure 2). The vertical axes indicate water depth. For Lake Zurich and Maggiore, spatio-temporal measurements are shown by contours. Dots indicate sampling depths and dates, shaded areas missing measurements. In Lake Maggiore, since total prokaryotic abundances were determined in the integrated epilimnetic (0–20 m) and hypolimnetic (20–350 m) samples (Figure 2), the averaged percentage for each layer was used to generate average CL500-11 abundances for epi- and hypolimnion.

**FIGURE 5:**
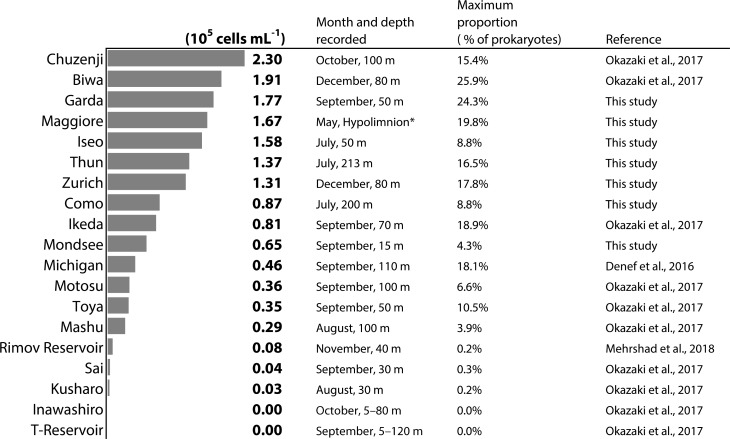
Maximum abundance of CL500-11 recorded by CARD-FISH analyses in each lake. ^∗^Bacterial abundance in the hypolimnion of Lake Maggiore was determined using an integrated sample from 20 to 350 m.

The vertical profile of temperature and dissolved oxygen indicated that the oxygenated (dissolved oxygen > 5 mg L^-1^) hypolimnion (the water layer below the thermocline) was present in all lakes, though the deeper hypolimnion (150–251 m) was anoxic in Lake Iseo and oxygen depletion in the bottom layer was observed in Lake Zurich and Mondsee (Figure 1). Total prokaryotic abundances ranged from 0.2 × 10^6^ (Lake Maggiore) to 5.2 × 10^6^ (Mondsee) cells mL^-1^ in the epilimnia, and 0.1 × 10^6^ (Lake Maggiore) to 3.2 × 10^6^ (Mondsee) cells mL^-1^ in the hypolimnia of the seven lakes (Figure 2). The CL500-11 cells detected by CARD-FISH were of the same size and shape (vibrioid, 1–2 μm length; Figure 3) as previously described (Okazaki et al., 2013, 2017; Denef et al., 2016; Mehrshad et al., 2018), indicating universality of their cell morphology. High proportions of CL500-11 were observed in the oxygenated hypolimnia of all investigated lakes; the maximum value recorded in each lake was 4.3% (Mondsee), 8.8% (Como and Iseo), 16.5% (Thun), 17.8% (Zurich), 19.8% (Maggiore), and 24.3% (Garda), resulted in their calculated abundances from 0.65 × 10^5^ (Mondsee) to 1.77 × 10^5^ (Garda) cells mL^-1^ (Figure 4). These are within the range of previously reported values in other lakes (Figure 5).

### The Oxygenated Hypolimnion as Preferred Habitat for CL500-11

The vertical distribution of CL500-11 showed a virtual absence in the epilimnion during the stratification period (Figure 4), thus further underpinning their prior adaptation to the hypolimnion environment. They were also not abundant in either anoxic bottom layers or in the transition zone between oxic and suboxic layers, as observed in Lake Zurich, Iseo, and Mondsee (Figures 1, 4), supporting the predictions from MAGs that CL500-11 are strict aerobes (Denef et al., 2016; Mehrshad et al., 2018). The time-series sampling in Lake Zurich and Maggiore revealed dynamic spatio-temporal successions of CL500-11 (Figure 4). Notably, the high-resolution sampling in Lake Zurich (fortnightly sampling of 9 depths) revealed that the water layers with absence and dominance of CL500-11 were clearly separated by the thermocline that was deepening with season (Figures 1, 4). This indicates that CL500-11 do not survive for long times in the epilimnion. Although the cause of CL500-11 mortality in the epilimnion is unknown, it might be possible that physico-chemical conditions there, such as high water temperature and light radiation, are lethal for these bacteria.

In Lake Biwa, CL500-11 abundances decreased below detection limit during the winter mixing period (Okazaki et al., 2013). On the contrary, the spatio-temporal profiles in Lake Zurich and Maggiore showed low but still detectable numbers (1.5–4.1% of all prokaryotes) of CL500-11 prevailed throughout the winter mixing (Figure 4), in line with reported CL500-11 at the end of the spring mixing period in the dimictic Lake Michigan (Denef et al., 2016). They might be present throughout winter mixing in Lake Garda as well, as a recent study detected *Anaerolineae*-related 16S rRNA sequences in the lake throughout the mixing period for two consecutive years (Salmaso et al., 2018). Since the abundance of CL500-11 showed decreasing trends during winter mixing (Figure 4), the mixed water column is likely a suboptimum habitat for them, and thus, their survival during winter mixing period may be a vital factor as it determines their initial abundance at the onset of the next stratification phase. It is notable that *in situ* metatranscriptome data from Lake Michigan showed that many CL500-11 genes, including rhodopsins and genes for oxidative stress response mechanisms, were expressed more in the surface water during the mixing period than in the hypolimnion (Denef et al., 2016). This suggests that CL500-11 can potentially front the mixing event and optimize their survival mechanisms until the onset of the next stratification phase. If the mixing disturbance is a non-preferred event for CL500-11, water depth might be one of the determinants for their abundance during the mixing period. Likely in deep lakes,—due to a higher volumetric hypolimnion-to-epilimnion ratio, the mixing disturbance is less intense, resulting in less exposure to light and higher concentrations of hypolimnion-originated dissolved substances and biota, including CL500-11 themselves. Indeed, positive correlations (Spearman’s coefficients, *p* < 0.05) were observed between CL500-11 cell density and the maximum depth or water volume of a lake (Table 2). This “dilution effect” might be one of the reasons for the absence of CL500-11 during the winter mixing period in Lake Biwa (Okazaki et al., 2013), as the lake has relatively shallow depth (73 m) at the sampling site, compared to those in Lake Maggiore (350 m), Zurich (136 m), Michigan (110 m), and Garda (350 m).

**Table 2:** The Spearman’s coefficients (ρ) and the corresponding *p*-values between the CL500-11 maximum abundance and environmental measurements in each lake.

	Surface area	Surface elevation	Water retention time	Maximum depth	Water volume	Hypolimnetic temperature during stratification	pH (min)	pH (max)	TN (min)	TN (max)	TP (min)^∗^	TP (max)
*p*-value	0.136	0.459	0.657	**0.025**	**0.034**	0.107	**0.004**	0.251	0.270	0.432	0.104	**0.022**
ρ	0.355	-0.181	0.109	0.512	0.488	0.381	0.631	0.277	0.284	0.204	0.385	0.520


*The original data used for the statistics are available in Supplementary Table S2. *p*-values lower than 0.05 are shown in bold. For pH, TN, and TP, minimum (min) and maximum (max) values recorded in each lake were separately used. ^∗^TP measurements below detection limits were replaced with the value of the detection limit.*

### Possible Substrates Supporting Growth of CL500-11

Despite the exclusive occurrence of CL500-11 in oxygenated hypolimnia, the result of the present study, together with previous reports of CL500-11 occurrence (Supplementary Table S2), revealed a broad habitat spectrum of CL500-11 (Table 3). CL500-11 have been detected in oxygenated hypolimnia of lakes and reservoirs located across the globe, with a variety in origin, trophic state, hypolimnetic temperature, surface area, depth, mixing regime, and water retention time (Table 3 and Supplementary Table S2). Moreover, most of the investigated environmental parameters, including trophic load of the lakes (i.e., total nitrogen and phosphorus), did not show significant correlations with CL500-11 abundances (Table 2). Indeed, the dominance of CL500-11 was observed in both ultra-oligotrophic Crater Lake (Urbach et al., 2001) and meso-eutrophic Lake Iseo (Figure 4). These facts suggest that substrates supporting growth of CL500-11 are ubiquitously present in oxygenated hypolimnia of freshwater systems.

**Table 3:** The range of environmental measurements of the lakes where CL500-11 were detected.


Region	Asia, Europe, North America, South America
Lake origin	Glacial, Caldera, Tectonic, Dammed, Artificial reservoir
Trophic state	Oligotrophic to meso-eutrophic
Surface area (km^2^)	2.06–82097
Surface elevation (m)	60–2357
Water retention time (y)	0.3–330
Maximum depth (m)	43–1637
Water volume (10^6^ m^3^)	34.5–23615000
Mixing regime	Dimictic, Monomictic, Meromictic^∗∗^
Occurrence of anoxic bottom layer	Yes and No
Hypolimnetic temperature during stratification^∗^ (°C)	3.8–10.9
pH^∗^	6.8–9.9
TN^∗^ (μg L^-1^)	60–2002
TP^∗^ (μg L^-1^)	2.55–45


*The data for each lake are available in Supplementary Table S2. ^∗^Lakes only with cloning/amplicon sequencing were not included for these parameters. ^∗∗^The upper hypolimnion was oxygenated in these meromictic lakes.*

High numbers of CL500-11 were observed in broad layers of hypolimnion; for instance, they accounted for >5% of all prokaryotes from 50 to 350 m in Lake Garda and from 50 to 213 m in Lake Thun (Figure 4). In line with previous observations (Okazaki et al., 2013, 2017), all of the observed CL500-11 cells were planktonic, i.e., not particle-associated (Figure 3). Since all samples were taken at pelagic stations, terrestrial input is likely less important for CL500-11 growth than autochthonous resources produced within the lake. Consequently, CL500-11 are likely depending on autochthonous DOM distributed in the whole hypolimnion. In freshwater systems, refractory DOM accumulating in the water column can be more than a 100 years old (Goldberg et al., 2015) and old substrates can still potentially be bioavailable (Guillemette et al., 2017). Assuming that DOM supporting CL500-11 growth would accumulate in the water column over a year, one may expect that lakes with longer water retention time would show higher abundances of CL500-11 due to higher concentrations of the DOM. However, no significant relationship between CL500-11 abundances and water retention times was detected (Table 2), i.e., high abundances were also observed in lakes with short water retention times, including Lake Zurich (1.4 years) and Thun (1.8 years) (Table 1 and Figure 4). It is thus likely that the resources for CL500-11 growth do not accumulate in the water column over a year but likely turnover within an annual timescale. This is in accordance with a stable isotope analysis in Lake Biwa (Maki et al., 2010) reporting that semi-labile autochthonous DOM, originally generated and accumulated in the epilimnion, was transported to the whole hypolimnion during winter mixing where it was slowly being consumed during the subsequent stratification period. Besides, CL500-11 were not abundant in two artificial reservoirs (Řimov and T) with water retention times of around 0.3 years (Figure 5 and Supplementary Table S2), supporting the hypothesis that a water retention time of more than a year is required to maintain a stable population of CL500-11.

The seasonal profiles of Lake Zurich (Figure 4) and Biwa (Okazaki et al., 2013) indicate that the abundance of CL500-11 increases toward the end of the stratification period, implying that their resources for growth are continuously supplied in the hypolimnion in these lakes till the end of the stratification period. These facts evoke an additional hypothesis that CL500-11 utilize substrates secondary produced *in situ* by microbial processes (Ogawa et al., 2001; Jiao and Zheng, 2011; Guillemette and del Giorgio, 2012). Indeed, studies have demonstrated that, similar to marine systems (Timko et al., 2015b; Shen and Benner, 2018), DOM is more photo-labile in the hypolimnion than in the epilimnion (Hayakawa et al., 2016; Yamada et al., 2018), suggesting that a part of hypolimnetic DOM is produced *in situ* and distinct from DOM produced and accumulated in the epilimnion, which likely consist of already-photobleached substrates such as carboxylic acids (Bertilsson and Tranvik, 2000). Correspondingly, a low number of transporters for carboxylic acids was found in CL500-11 MAGs compared to other freshwater microbes (Denef et al., 2016), pointing to a non-preferred utilization of these photobleached compounds. On the other hand, CL500-11 MAGs harbor a high number of transporters for di- and oligopeptide, as well as transporters for spermidine/putrescein, and branched-chain amino acids (Denef et al., 2016; Mehrshad et al., 2018). Such nitrogen-rich, protein-like DOM can be originated from remnants of bacterial cell walls (McCarthy et al., 1998; Nagata et al., 2003) and membranes (Tanoue et al., 1995), and consumption of such DOM by the microbial community in the oxygenated hypolimnion have been suggested by DOM profiling by stoichiometry (Kim et al., 2006) and by excitation and emission matrices (Thottathil et al., 2013; Hayakawa et al., 2016) in Lake Biwa. One CL500-11 MAG also included a transporter for *N*-acetylglucosamine, a breakdown product of bacterial cell walls (Denef et al., 2016; Mehrshad et al., 2018). Moreover, a study has reported 16S rRNA gene sequences of CL500-11 from an actively growing prokaryotic community in *N*-acetylglucosamine-enriched lake water (Tada and Grossart, 2014). These data collectively lead to the speculation that CL500-11 are scavenging cell wall compounds or other cellular remnants released *in situ* by grazing or viral lysis of other prokaryotes (Nagata et al., 2003; Middelboe and Jørgensen, 2006; Eckert et al., 2013), which can be continuously produced in the water column of hypolimnion.

### Potential Limiting Factors for the Distribution of CL500-11

Besides the two artificial reservoirs discussed above, low abundances of CL500-11 in the oxygenated hypolimnion during the stratified period were also found in previously investigated Lake Kussharo and Inawashiro (Okazaki et al., 2017) (Figure 5), despite their relatively high maximum depth (118 and 94 m, respectively), large surface area (79.6 and 103.3 km^2^), and long water retention time (12 and 5.4 years) (Supplementary Table S2). It is notable that these lakes receive inflows influenced by volcanic activities and were acidic (pH < 5.0) until 30 years ago, followed by neutralization with pH ranges of 7–7.5 and 6.6–7, respectively, to date (Oyama et al., 2014; Sutani et al., 2014). Further, we have recently noticed an absence of CL500-11 in the water column of Lake Tazawa, a deep (423 m), slightly acidic (pH < 6) oligotrophic freshwater lake, which receives acidic volcanic inflow as well (Okazaki et al., unpublished data). The statistical test also supported a positive relationship (*p* = 0.004) between CL500-11 abundances and minimum recorded pH in a lake (Table 2). Although the direct factor affecting CL500-11 in low pH condition is unknown, it is likely that they cannot survive in lakes affected by acidic volcanic inflow. In addition to anion and cation concentrations, pH may affect photo-liability and colloidal size of DOM (Pace et al., 2012; Timko et al., 2015a). These factors may be critical for CL500-11 survival, in accordance to other studies that identified pH as master driver for the distribution of different microbial lineages (Lindstrom et al., 2005; Newton et al., 2011).

In Lake Maggiore, severe drops in the total prokaryotic abundance were observed in September in the hypolimnion and in November in the epilimnion (Figure 2), which resulted in a drastic decline of the CL500-11 population in September as well (Figure 4). Such dynamics in abundance might be attributable to top-down control, namely grazing and viral lysis, yet unfortunately, no data is available for the viral and predators’ communities during the study period. Recent observations reported hypolimnion-specific heterotrophic nanoflagellate communities (Mukherjee et al., 2015, 2017) that might be grazing on CL500-11. Larger mixotrophic flagellates and ciliates may also be the major predator of CL500-11, as they can efficiently graze bacterioplankton of this size as well (Posch et al., 1999). It is likely that CL500-11 are also lysed by specific viruses, since virus-host interactions are more specific than size-selective prey-predator interactions. The diversity of grazers and viruses in oxygenated hypolimnia remains largely unexplored, and the main mortality agents for CL500-11 are yet-to-be identified. Future studies should pay more attention to understand mechanisms behind CL500-11 population dynamics and the turnover of organic matter via CL500-11.

## Conclusion

Overall, the quantitative investigation of CL500-11 in perialpine lakes further endorsed ubiquity and quantitative significance of this bacterioplankton lineage in global deep freshwater systems (Figures 4, 5 and Table 3). Our results as well as metagenomic and geochemical insights from literatures allowed to hypothesize possible characteristics of substrates supporting CL500-11 growth, most likely nitrogen-rich autochthonous DOM ubiquitously available in the hypolimnion that may turnover within an annual timescale. CL500-11 may either directly utilize DOM derived from the epilimnion transferred by winter vertical mixing, or depend on DOM secondary produced *in situ*, for example, cellular debris from other microbes. We further suggest that shallow depth, low pH, and short water retention time would limit CL500-11 dominance. However, eco-physiological mechanisms and consequences of these findings and assumptions are still mostly unknown or lack direct evidences, and there still remain unexplored factors potentially controlling CL500-11 dynamics such as grazing and viral infection. These subjects need further verification in future studies.

Not only their importance in ecosystem and biogeochemical cycling, but also their evolutionary and biogeographic backgrounds are intriguing topics for debate, given that CL500-11 is the sole bacterioplankton lineage affiliated with *Chloroflexi* dominating in freshwater systems and exclusively inhabiting the oxygenated hypolimnion. Although the ecology of CL500-11 has been gradually understood by the growing number of researches, there may be still many remarkable characteristics yet-to-be discovered. For instance, a recent metagenomic study revealed that CL500-11 are present even in the brackish Caspian Sea, and identified at least three species in four investigated lakes based on average nucleotide identities of MAGs, for which a candidate genus Profundisolitarius was proposed (Mehrshad et al., 2018). Continuous efforts should be made to uncover the ecology of this enigmatic bacteria by further field explorations and meta-omics surveys, along with the ultimate challenge toward their isolation and cultivation (Salcher and Šimek, 2016).

## Author Contributions

YO conceived the study. MS and CC collected the samples and field measurements. YO and MS performed CARD-FISH. YO, MS, CC, and SN wrote the manuscript.

## Conflict of Interest Statement

The authors declare that the research was conducted in the absence of any commercial or financial relationships that could be construed as a potential conflict of interest.

## Footnotes

1 https://www.arb-silva.de/search/testprobe/

## Supplementary Material

Table S1 List of the 60 sequences in the SILVA SSU 132 Parc database that perfectly match the probe CLGNS_584. Nucleotide identities to the two CL500-11 sequences in the SILVA SSU 132 Ref NR99 database (AF316759 and HM856384) are shown in the second and third columns. The other columns are retrieved from the output of the TestProbe 3.0 tool.

Table S2 Summarized information for lakes where CL500-11 were investigated or detected. The first sheet contains lakes where CL500-11 have been enumerated with CARD-FISH, and the second sheet includes lakes where 16S rRNA sequences of CL500-11 have been retrieved. The references for the data are summarized in the third sheet.
